# Overlapping and Distinct Physical and Biological Phenotypes in Pure Frailty and Obese Frailty

**DOI:** 10.1042/BSR20240784

**Published:** 2024-11-06

**Authors:** Fujue Ji, Ji Hyun Park, Hyeonseung Rheem, Jong-Hee Kim

**Affiliations:** 1 Major in Sport Science, Division of Sport Industry and Science, College of Performing Arts and Sport, Hanyang University, 222 Wangsimni-ro, Seongdong-gu, Seoul, Republic of Korea; 2 BK21 FOUR Human-Tech Convergence Program, Hanyang University, 222 Wangsimni-ro, Seongdong-gu, Seoul 04763, Republic of Korea

**Keywords:** frailty, obesity, phenotype

## Abstract

Background: Pure frailty and obese frailty are common types of frailty syndrome. However, the overlapping and distinct characteristics between pure frailty and obese frailty remain unclear. This study aims to reveal the overlapping/distinct physical and biological phenotypes of pure frailty and obese frailty, providing theoretical support for their prevention, diagnosis, and treatment.

Method: Mice were fed either a normal or high-fat diet and assessed at 20 months of age. They were assigned to one of the four groups: control, obesity, pure frailty, and obese frailty. Grip strength, walking speed, physical activity, endurance, and body weight were measured to determine pure frailty and obese frailty. Physical and biological phenotypes were assessed.

Results: Distinct physical phenotypes were observed between pure frailty and obese frailty in terms of body weight, lean mass, fat mass, fat mass in tissue, grip strength, endurance, and physical activity, while walking speed overlapped. In biological phenotypes, levels of Smad2/3, FoxO3a, P62, LAMP-2, and cathepsin L expression were distinct, while AKT, p-AKT, mTOR, p-mTOR, p-Smad2/3, p-FoxO3a, Beclin-1, ATG7, and LC3 overlapped.

Conclusion: Distinct physical phenotypes observed in obese frailty are primarily attributable to the effect of obesity, with further impairment of muscle function resulting from the combined effects of frailty syndromes and obesity. Pure frailty and obese frailty share overlapping biological phenotypes, particularly in the regulation of muscle protein synthesis. Moreover, the interaction between obesity and frailty syndromes gives rise to both overlapping and distinct biological phenotypes, especially in the regulation of specific degradation signaling proteins.

## Introduction

Frailty syndromes, which increase with age, pose a major challenge to public health [1]. This common geriatric syndrome can occur independently of specific diseases and signifies a marked health decline in older adults [2–4]. Frailty syndromes elevate the risk of adverse outcomes, including falls, disability, hospitalization, and mortality [5,6]. While weight loss is a major contributing factor to frailty syndrome [7], many clinical [8–10] and preclinical studies [11,12] have shown that obesity is also strongly associated with frailty syndromes. Both low body weight and obesity in older adults are linked to frailty syndromes [2,13], with 80.2% of frailty cases in older adults involving underweight individuals in one study [13] and 64.4% involving obesity in another study [2].

Body weight changes associated with frailty syndrome are closely tied to decreased functional capacity and quality of life, manifesting as alterations in physical phenotypes at diagnosis [14]. Both pure frailty, which is typically accompanied by weight loss, and obese frailty lead to diminished physical function, resulting in a significant decline in physical phenotype indices. However, the negative health effects of pure frailty and obese frailty due to weight changes differ [8,12,15,16]. Obese frailty does not cause unintentional weight loss but can lead to complications like diabetes and hypertension [8,12]. In contrast, weight loss due to pure frailty can lead to cachexia [15,16]. Therefore, the connections and distinctions between pure frailty and obese frailty require clear definition through systematic study, focusing on both overlapping and distinct physical and biological phenotypes.

Currently, the most well-known physical phenotype difference between pure frailty and obese frailty is body weight. Beyond weight changes, pure frailty leads to lower physical phenotypes indices like endurance, physical activity, strength, and walking speed [17,18]. Obesity can have varied effects on these indices; for instance, it may increase strength [19,20]. Hence, undiscovered overlapping and distinct physical phenotypes for pure frailty and obese frailty likely exist. Additionally, investigating the biological phenotypes of pure frailty and obese frailty is crucial [21]. Biological phenotypes provide objective and measurable health indicators and can precede physical phenotypes and clinical manifestations [22]. Because changes in biological phenotypes can precede debilitating physical phenotypes and clinical manifestations [23], they offer a current picture of disease progression. Research on the biological phenotypes of pure frailty is limited to findings of increased inflammation [24,25], immune disorders [26–28], and oxidative stress [17,29,30]. Research on the biological phenotypes of obese frailty remains sparse. Obese frailty involves pathophysiologic mechanisms common to both frailty syndrome and obesity (e.g., chronic inflammation and oxidative stress), which may reinforce each other [31–34]. These findings suggest that overlapping and distinct biological phenotypes likely exist for pure frailty and obese frailty.

Obese frailty may not be simply a combination of frailty syndrome and obesity; rather, it may involve complex pathogenic mechanisms and cellular signaling pathways that interact. A better understanding of the phenotypic differences between pure frailty and obese frailty would have significant clinical implications. Recognizing distinct physical and biological phenotypes allows for the development of targeted interventions tailored to each condition, thereby improving treatment and prevention strategies. Additionally, differentiating between pure frailty and obese frailty enhances diagnostic accuracy, leading to more effective management plans for health declines in older adults. Furthermore, this understanding supports a comprehensive approach to health management, emphasizing the need to address both weight management and muscle function. These insights are crucial for developing interventions that cater specifically to patients with pure frailty, obese frailty, or both, ultimately improving clinical outcomes for frailty syndromes.

## Method

### Animals and diets

Male C57BL/6N mice (14 months old, n = 60) were divided into two groups: normal diet (ND, n = 30) and high-fat diet (HF, n = 30). The ND group was fed a standard diet (AL-C, Chow, Teklad Global 2018, Envigo Inc.), while the HF group received a high-fat diet (45% fat; AL-H, D12451, Research Diets Inc.) for 6 months until they reached 20 months of age (Table 1). Environmental conditions were controlled with a room temperature of 22 ± 2 °C, humidity of 50–60%, and a 12/12-h light/dark cycle. Mice had ad libitum access to water and activity. All procedures were approved by the Institutional Animal Care and Use Committee (IACUC) of Hanyang University (HYU 2021-0066A).

**Table 1:** Diet composition

Group	ND		HF	
Diet	Teklad Global 2018		D12451	
	g%	kcal%	g%	kcal%
Protein	18.4	30	24	20
Carbohydrate	44.2	50	41	35
Fat	6	20	24	45
Total		100		100
Kacl/gm	3.1		4.73	

### Determination of pure frailty and obese frailty

**Figure 1:**
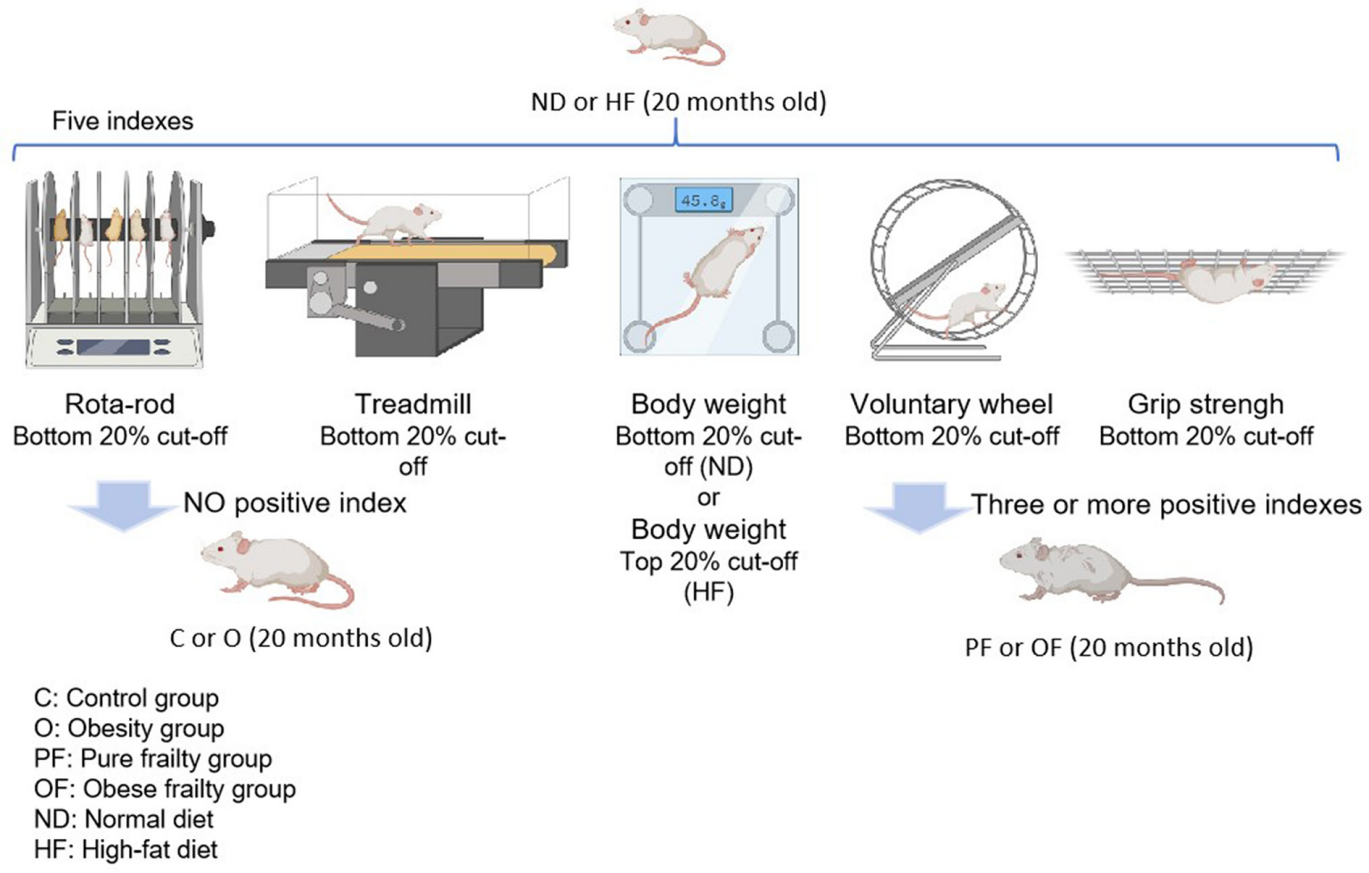
The pure frailty and obese frailty screening process

At 20 months, mice were screened for pure frailty and obese frailty using modified criteria based on prior research [11,17,35]. Briefly, three or more of the five physical phenotype indices (walking speed, endurance capacity, body weight, physical activity, and grip strength) being positive qualified mice as having pure frailty or obese frailty. Following the percentiles used by Baumann et al. [11], in ND, five index measurements within the bottom 20% were considered positive for pure frailty; in HF, the same five index measurements were used as ND, but those in the top 20% for body weight were considered positive for obese frailty (Figure 1). Finally, the mice were divided into four groups: control (C, n = 8), pure frailty (PF, n = 7), obesity (O, n = 7), and obese frailty (OF, n = 7).

### Physical phenotype assessment

#### Rota-Rod test

According to Graber et al. [36], mice were tested for walking speed by Rota-Rod. The adaptation period was performed during the first 3 days of the official test. The adaptive test exercise consisted of 5 rpm for 1 min, once per day, for 3 days. The official test used the acceleration mode, with the speed increasing from 5 to 50 rpm over a 5-min period (Model 76-0770, Harvard Apparatus Inc., Holliston, MA, USA). The latency to fall from the device was recorded. Mice were tested three times, with a 10-min interval between each test. The best performance out of the three trials was used as the outcome measure.

#### Treadmill test

A protocol for testing endurance capacity in mice using a treadmill was developed by Marcaletti et al. [37]. Adaptation was performed 3 days before the official test. Adaptive test exercise: 5 cm/s, 5 min, once per day, for 3 days, and 0° slope. In the official test, mice ran on the treadmill as it accelerated by 5 cm/s every minute starting at 5 cm/s, with a 0° slope. The trial was terminated after the mice had touched the shock pad (set at 0.6 mA) three times.

#### Body weight

The use of Dual Energy X-ray Absorptiometry (DEXA) to accurately evaluate mouse body composition has been widely accepted [38,39]. Mice were anesthetized and placed into an InAlyzer DEXA (Micro Photonics Inc., PA, USA) for analysis of body composition, including body weight (g), fat mass (g), lean mass (g), and fat mass in tissue (%).

#### Voluntary wheel test

The physical activity of mice can be tested using a voluntary wheel [40]. In this study, the running distance was measured by a voluntary wheel (MAN86130, Lafayette Instrument Company, Lafayette, IN 47904, USA), with each rotation of the wheel corresponding to a distance of 0.4 m. The average running distance over a 5-day period was recorded for each mouse.

#### Grip strength test

The grip strength of the mice was evaluated using the inverted-cling grip test [41]. Adaptations were conducted prior to the start of the official test. The adaptive inverted-cling grip test was performed once a day for 3 days. During the official test, the mice were placed in the center of the wire mesh screen, and a stopwatch was started. The screen was rotated to an inverted position over 2 s, with the mouse’s head declining first. The screen was held steadily 40–50 cm above a padded surface. The time when the mouse fell off was recorded. The measurement was repeated three times with test intervals of 10 min, and the maximum reading was recorded.

### Biological phenotype assessment

Mice were anesthetized with an intraperitoneal injection of 40 mg/kg ketamine and 0.8 mg/kg medetomidine. Following anesthesia, the mice were killed by cervical dislocation. The gastrocnemius muscles were lysed and homogenized using an EzRIPA Lysis Kit (WSE-7420, ATTO), containing protease and phosphatase inhibitors to maintain protein integrity. The samples were centrifuged at 13 000 g for 10 min at 4°C to pellet cell debris, and the supernatant containing soluble proteins was collected from the lysate. Protein concentration was determined using a PierceTM Bicinchoninic Acid (BCA) Protein Assay Kit (23225, Thermo Scientific). Electrophoresis was performed using a 4% stacking gel and a 12% separating gel. The protein samples were mixed with a 5× sample buffer (ELPis, EBA-1052) containing 312.5 mM Tris-HCl (pH 6.8), 50% glycerol, 5% SDS, 5% β-mercaptoethanol, and 0.05% bromophenol blue. Equal amounts of protein (45 μg per lane) were loaded onto the gel along with a molecular weight marker (GENDEPOT, P8502) for size estimation. SDS-PAGE was performed under constant voltage (100 V for 120 min) until the dye front reached the bottom of the gel. The running buffer contained Tris base (3 g), glycine (14.4 g), SDS (1 g), and distilled water (1 l). Following electrophoresis, the transfer sandwich was assembled with the gel, nitrocellulose membrane (0.2 µm, BIO-RAD, cat#1620112), and filter paper soaked in transfer buffer (Tris base 3.03 g, glycine 14.4 g, distilled water 800 ml, and methanol 200 ml). Electrotransfer was performed at a constant current (30 V) for 12 h at 4°C. After the transfer, the membrane was incubated in blocking buffer (5% non-fat milk in TBST) for 1 h at room temperature. TBST contained 10 mM Tris, 150 mM NaCl, and 0.1% Tween-20; pH 7.6. The membrane was washed with TBST to remove excess blocking buffer (three washes of 5 min each). The primary antibody (dilution as recommended, see Table 2) was diluted in blocking buffer and the membrane was incubated with the primary antibody overnight at 4°C with gentle agitation. The membrane was then washed with TBST to remove unbound primary antibody (three washes of 5 min each). The secondary antibody (dilution as recommended, see Table 2) conjugated to horseradish peroxidase was diluted in blocking buffer and the membrane was incubated with the secondary antibody for 1.5 h at room temperature with gentle agitation. The membrane was washed with TBST to remove unbound secondary antibody (three washes of 5 min each). Protein bands were visualized using an ECL (Cytiva, RNP2106) chemiluminescence substrate and images were captured using a chemiluminescence imaging system (BIO-RAD XRS+). Band intensities were quantified using ImageJ software. Protein loading was confirmed and normalized using Ponceau S staining.

### Venn diagrams

Venn diagrams were created using Python to quantify the overlapping physical and biological phenotypes of pure frailty and obese frailty. The Python code used to generate these Venn diagrams is included in the supplementary files (S1).

**Table 2:** Antibody

Antibody	Source	Vender	Catalog	Dilution
p-mTOR^Ser2448^	Rabbit lgG	Cell signaling	#5536	1:1000
mTOR	Rabbit lgG	Cell signaling	#2983	1:500
p-AKT^Ser473^	Rabbit lgG	Cell signaling	#4060	1:1000
AKT	Rabbit lgG	Cell signaling	#9272	1:500
p-Smad2^Ser465/467^/3^Ser423/425^	Rabbit lgG	Cell signaling	#8828	1:1000
Smad2/3	Mouse lgG	Santa Cruz	sc-133098	1:1000
p-FoxO3a^Ser253^	Rabbit lgG	Cell signaling	#13129	1:500
FoxO3a	Rabbit lgG	Cell signaling	#12829	1:500
Beclin-1	Rabbit lgG	Cell signaling	#3495	1:500
ATG7	Rabbit lgG	Cell signaling	#8558	1:1000
p62	Rabbit lgG	Cell signaling	#23214	1:1000
LC3	Rabbit lgG	Sigma-Aldrich	L7543	1:500
LAMP-2	Rabbit lgG	Invitrogen	#PA1-655	1:500
Cathepsin L	Rabbit lgG	Abcam	ab133641	1:500
Goat anti Rabbit	Goat lgG	Invitrogen	#G-21234	1:5000
m-IgG Fc	–	Santa Cruz	sc-525409	1:5000

### Statistical analyses

Analyses were performed using GraphPad Prism (version 9) software. A two-way analysis of variance was used to compare the mean differences among groups. *P*<0.05 was considered statistically significant. Significant differences are denoted by asterisks: *P*<0.05 (*), *P*<0.01 (**), *P*<0.001 (***), and *P*<0.0001 (****). All values are presented as mean ± SEM.

## Results

### Pure frailty and obese frailty: overlapping and distinct physical phenotypes

**Figure 2:**
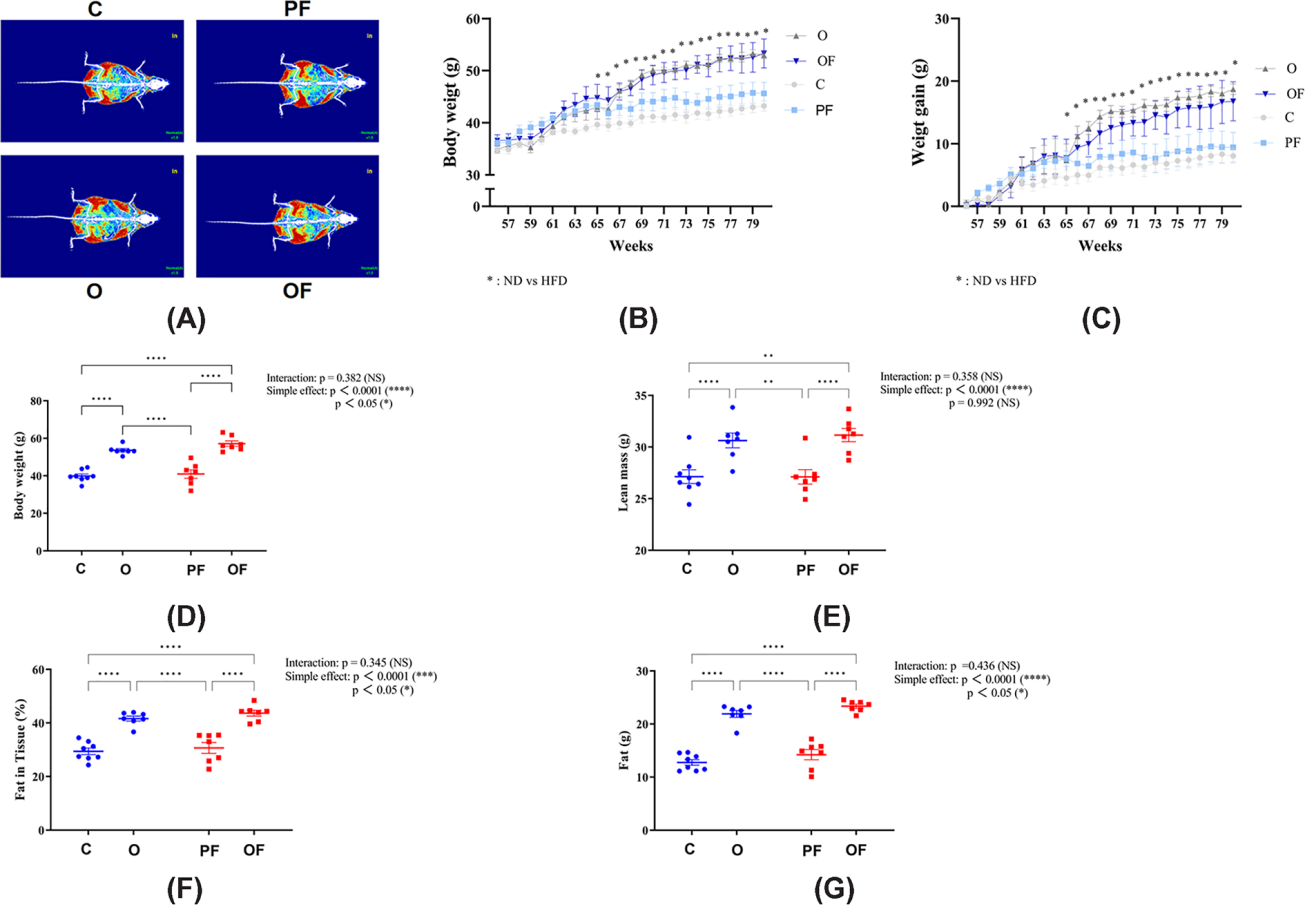
Changes in the physical phenotype of body composition (**A**) Representative DEXA scan images, and skeletal muscle, fat tissue, and bone are shown in blue, red, and white, respectively. (**B**) Weekly body weight changes (group × weeks): the following symbols indicate differences: C and PF vs. O and OF (*, 56–74 weeks). (**C**) Weekly body weight gains (group × weeks): the following symbols indicate differences: C and PF vs. O and OF (*, 56–74 weeks). (**D–G**) Quantitative data on final body weight, lean mass, fat mass, and fat mass in tissue, respectively, were acquired by DEXA scanning. Significant differences are denoted by an asterisk:* P*<0.05 (*), *P*< 0.01 (**), *P*<0.001 (***), and *P*<0.0001 (****). All values are presented as mean ± SEM. C, control (20 months, n = 8); PF, pure frailty (20 months, n = 7); O, obesity (20 months, n = 7); and OF, obese frailty (20 months, n = 7).

The results demonstrated no significant differences in body composition under the same dietary conditions (*P*>0.05, Figure 2D–G). This finding suggests that body composition alone cannot distinguish between control and pure frailty in the normal diet group, nor between obesity and obese frailty in the high-fat diet group. However, compared with the control and pure frailty groups, the obesity and obese frailty groups exhibited significantly higher body weight, lean mass, fat mass, and fat mass in tissue (*P*<0.05, Figure 2D–G). These findings suggest that body weight, lean mass, fat mass, and fat mass in tissue are a distinct physical phenotype of body composition between pure frailty and obese frailty.

**Figure 3:**
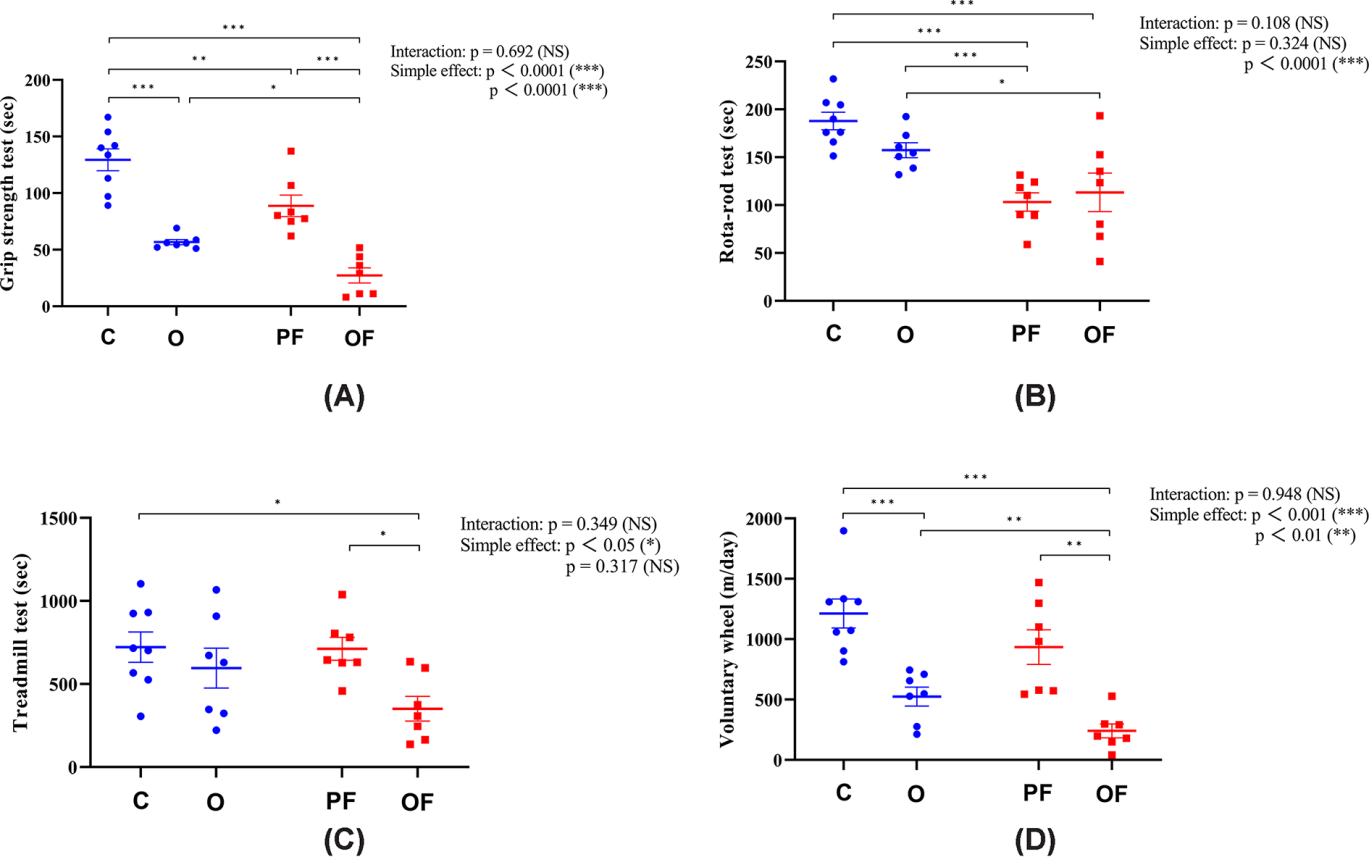
Physical phenotype tests (**A**) Grip strength test. (**B**) Rota-rod test (walking speed test). (**C**) Treadmill test (endurance capacity test). (**D**) Voluntary wheel test (physical activity test). Significant differences are denoted by an asterisk: *P*<0.05 (*), *P*<0.01 (**), and *P*<0.001 (***). All values are presented as mean ± SEM. C, control (20 months, n = 8); PF, pure frailty (20 months, n = 7); O, obesity (20 months, n = 7); and OF, obese frailty (20 months, n = 7).

The physical phenotype tests revealed that pure frailty significantly reduced grip strength and walking speed (*P*<0.05, Figure 3A and B). Obesity led to significant decreases in grip strength and physical activity (*P*<0.05, Figure 3A,C, and D). Compared with pure frailty, obese frailty resulted in further declines in grip strength, endurance, and physical activity (*P*<0.05, Figure 3A,C,and D). These findings suggest that the overlapping physical phenotype between pure frailty and obese frailty is reduced walking speed, while the distinct physical phenotype associated with obese frailty includes additional reductions in grip strength, endurance, and physical activity.

### Pure frailty and obese frailty: overlapping and distinct biological phenotypes

**Figure 4:**
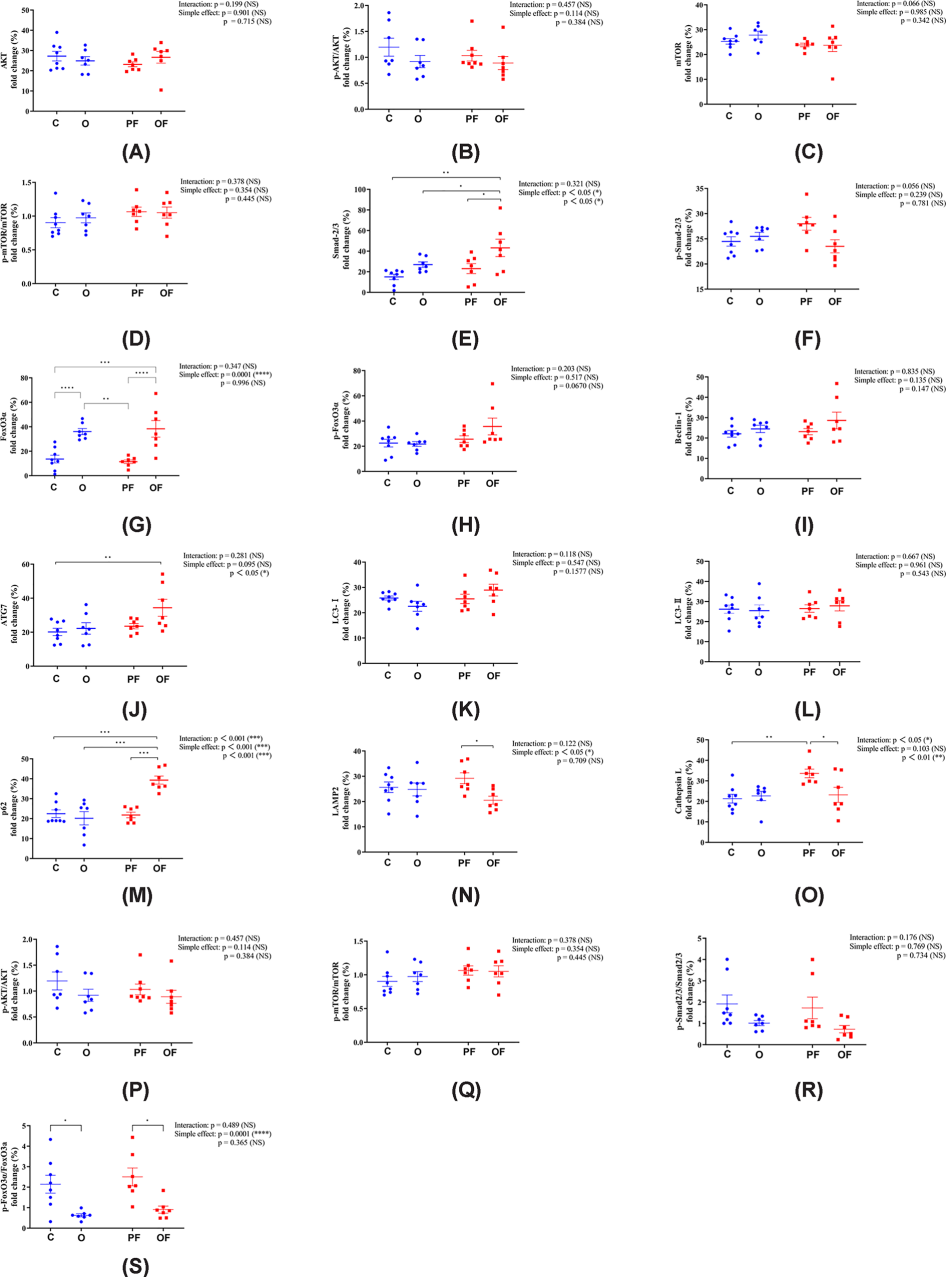
Biological phenotype change in skeletal muscle protein synthesis and degradation signaling pathway (**A,B**) AKT and p-AKT^Ser473^ expression. (**C,D**) mTOR and p-mTOR^Ser2448^ expression. (**E,F**) Samd2/3 and p-Samd^Ser465/467/3Ser423/425^ expression. (**G,H**) FoXO3a and p-FoXO3a^Ser253^ expression. (**I**) Beclin-1 expression. (**J**) ATG7 expression. (**K,L**) LC3-I and LC3-II expression. (**M**) P62 expression. (**N**) LAMP2 expression. (**O**) Cathepsin L expression. (**P**) p-AKT/AKT ratio. (**Q**) p-mTOR/mTOR ratio. (**R**) p-Smad2/3/Smad2/3 ratio. (**S**) p-FoxO3a/FoxO3a ratio. Significant differences are denoted by an asterisk: *P*<0.05 (*), *P*<0.01 (**), and *P*<0.001 (***). All values are presented as mean ± SEM. C, control (20 months, n = 8); PF, pure frailty (20 months, n = 7); O, obesity (20 months, n = 7); and OF, obese frailty (20 months, n = 7)

The results showed that under the same dietary conditions, pure frailty decreased the expression of p-AKT^Ser473^ in the skeletal muscle protein synthesis pathway, while obese frailty did not (*P*>0.05, Figure 4B). No significant differences in skeletal muscle protein synthesis signaling pathway were found between pure frailty and obese frailty (*P*>0.05, Figure 4A–D). In the protein degradation signaling pathway, compared with pure frailty, obese frailty resulted in significant increases in Smad2/3, FoxO3a, and P62 expression (*P*<0.05, Figure 4E,G,and M) and significant decreases in LAMP2 and cathepsin L expression (*P*<0.05, Figure 4N and O). The overlapping biological phenotypes of pure frailty and obese frailty included similar expression of AKT, p-AKT^Ser473^, mTOR, p-mTOR^Ser2448^, p-Smad2^Ser465/467/3Ser423/425^, p-FoxO3a^Ser253^, Beclin-1, ATG7, and LC3-I/II. Obese frailty led to significant increases in Smad2/3, FoxO3a, and P62 expression levels and significant decreases in LAMP2 and cathepsin L.

### Quantification of overlapping and distinct features

**Figure 5:**
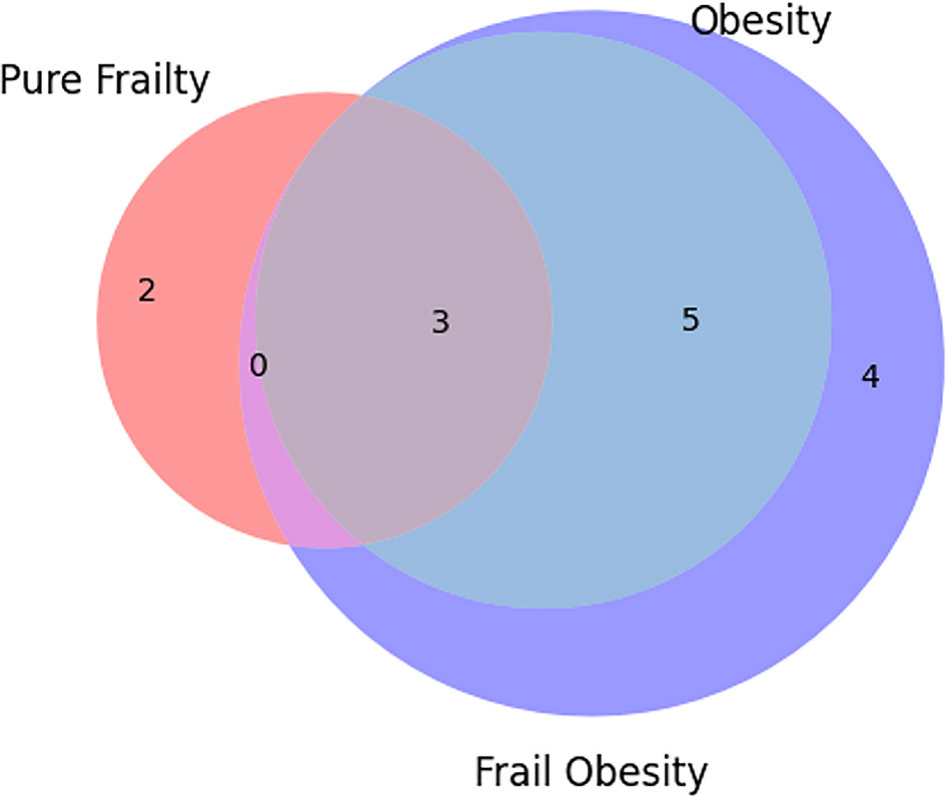
Overlapping and distinct features among obesity, pure frailty, and obese frailty All features of pure frailty are shown in pink, all features of obesity are shown in blue, and all features of obese frailty are shown in purple. The overlapping areas of the circles represent overlapping features, while the non-overlapping areas represent distinct features. The numbers indicate the quantity of overlap. This Venn diagram was created using Python.

Through experiments and statistical analysis, we identified three overlapping features among obesity, pure frailty, and obese frailty: low grip strength, reduced walking speed, and decreased p-AKT expression. These overlapping features were also observed between pure frailty and obese frailty, as well as between pure frailty and obesity. Obesity and obese frailty share five overlapping features: low grip strength, reduced walking speed, low physical activity, decreased p-AKT expression, and high FoxO3a expression. Pure frailty has two distinct features: low physical activity and reduced cathepsin L expression. Obese frailty exhibits four distinct features: reduced endurance, increased Smad2/3 expression, elevated ATG7 expression, and higher p62 expression. In contrast, obesity does not exhibit any distinct features compared with pure frailty. There are no features that are unique to obesity that overlap with both pure frailty and obese frailty Figure 5.

## Discussion

In this study, we developed mouse models for pure frailty and obese frailty by modifying existing methods [11,12,42]. Using normal and high-fat diets, we successfully screened these models to investigate their overlapping/distinct physical and biological phenotypes. To the best of our knowledge, this is the first study to comprehensively explore these phenotypes in the context of pure frailty and obese frailty. These findings represent a major advancement in our understanding of the phenotypic distinctions between pure frailty and obese frailty, which could inform more targeted approaches to diagnosis, treatment, and prevention.

### Appropriateness of mouse models

We conducted interventions on 14-month-old mice for 6 months, killing them at 20 months of age. Mice at 14 months of age are approximately equivalent to humans aged 35–45 years, and at 20 months, their age corresponds to a human aged 55–65 years [43]. Research indicates that the prevalence of obesity significantly increases after the age of 40 [44], while the incidence of frailty syndrome rises markedly after the age of 60 [45,46]. In our study, the mice were placed on a high-fat diet beginning at 14 months and were killed at 20 months. This experimental design models the progression of continuous obesity starting around age 40 in humans, leading to obesity or obese frailty by around age 60. Furthermore, since frailty syndrome is believed to be reversible, this timeframe provides an adequate window to explore potential life-changing interventions.

Current screening methods for pure frailty in mouse models are based on the criteria established by Fried et al. [42] with slight modifications. Liu and colleagues [35] used 27–28-month-old mice to identify pure frailty by assessing endurance, walking speed, grip strength, and physical activity. Building on Liu and colleagues’ [35] findings, Baumann and co-workers [47] examined the onset and progression of frailty syndrome using 13-month-old mice, adding a criterion for overweight rather than unintentional weight loss. Another study that screened for pure frailty in mice used a similar weight loss characterization to Fried et al. [17]. Gomez-Cabrera and colleagues [12] conducted experiments with 3-month-old mice, considering a weight loss of over 5% during aging as a positive indicator of frailty syndrome. However, our data (Figure 2B and C) did not show a consistent trend in weight loss with age, as observed by Gomez-Cabrera and colleagues [12]. Body weight changes in aging mice can be influenced by factors like environment [48] and diet [49]. Additionally, data from the Jackson Laboratory indicate that male C57BL/6N mice tend to gain weight with age under normal diet conditions. This age-related weight gain contradicts the typical characteristics of pure frailty. To address these variations, we adopted the bottom 20% of body weight as a cut-off point for identifying pure frailty and conducted weekly weight evaluations to capture more accurate changes. The 20% cut-off has been widely used and recognized in the diagnosis of frailty syndrome in both humans and mice [11,17,50–52]. For a detailed explanation of the use of the 20% cut-off point, refer to the comprehensive review by Thompson et al. [52].

Currently, there are no standardized screening criteria for obese frailty mouse models. In this study, obese frailty mice were identified using a high-fat diet-induced obesity model, confirmed by DEXA analysis. After 6 months on a high-fat diet, body weight, lean mass, fat mass, and fat mass percentage significantly increased, consistent with the characteristics of the established obesity model [38,39,53,54]. The top 20% of body weight among obese mice was used as a positive marker for obese frailty in this study. High body weight negatively impacts longevity across various species [55]. For instance, Ames dwarf mice, characterized by low body weight, have extraordinarily long lifespans [56,57], while calorie restriction, which lowers body weight, has been shown to increase lifespan in a wide range of mammals [58,59]. Our DEXA analysis indicated that increased fat mass contributed to the higher body weights observed. Epidemiological data show a strong positive correlation between fat mass and mortality [60,61]. Contrary to the traditional view of frailty as a ‘wasting disease’, obesity increases the risk of frailty syndromes [62,63]. Limited research has found that cardiovascular issues are more severe in patients with high body weight frailty syndromes [64]. Moreover, obesity in older adults is associated with increased risk of impaired physical functioning [65], which is strongly linked to frailty syndromes [66]. Finally, obesity also reduces anti-inflammatory factors [67,68] and stress resistance [69,70], further contributing to the development of frailty syndromes.

### Overlapping and distinct physical phenotypes in pure frailty and obese frailty

Our data showed that neither pure frailty nor obese frailty significantly altered body composition compared with their respective counterpart under the same dietary conditions (Figure 2D–G). In contrast, Baumann et al. found that healthy mice had lower body weight and fat mass compared with pure frailty, as they used the top 20% of body weight as a positive marker [47]. Gomez-Cabrera et al. used an unintentional 5% reduction in body weight as a positive marker, finding that healthy mice had significantly higher body weights than those with pure frailty [12]. The discrepancy in results between prior studies may be attributed to the differing criteria employed in screening for pure frailty. Instead of the upper 20% or 5% loss [12,47], we selected the bottom 20% of body weight as a positive marker of pure frailty. Our findings were corroborated by a cross-sectional study (n = 656), which found no significant difference in body weight between individuals with pure frailty and healthy controls [71]. Another study also reported no significant difference in fat mass between pure frailty and healthy controls [72]. These results suggest that pure frailty may not significantly affect body composition, which may be related to our criteria, where weight was considered a factor but not the sole determinant.

There is a notable research gap concerning the body composition of obese frailty, a comorbidity of frailty syndromes and obesity. Our data revealed that individuals with obese frailty exhibited greater body weight, lean mass, fat mass, and fat mass in tissue compared with those with pure frailty, but no significant differences were observed when compared with those with obesity (Figure 2D–G). Previous studies [73–75], along with our findings, indicate that obesity alone can lead to significant increases in these metrics, suggesting that differences in body composition between pure frailty and obese frailty may be primarily attributable to the effects of obesity.

Walking speed is an overlapping physical feature of pure frailty and obese frailty. Our data showed that both pure frailty and obese frailty resulted in reduced walking speed compared with the control and obesity groups. However, there were no significant differences between control and obesity groups, nor were frail and obese frailty. While obesity has often been associated with reduced walking speed [76–79], some studies [80–82] have reported that not all cases of obesity lead to reduced walking speed in rodents, which supports our findings. The reasons for these inconsistent findings remain unclear. In contrast with the uncertain effects of obesity on walking speed, the negative impact of frailty syndromes and aging on walking speed has been widely documented [12,35,47]. Therefore, the reduced walking speed observed in both pure frailty and obese frailty may be attributed more to frailty syndromes’ association with aging rather than obesity.

Distinct physical phenotypes between pure frailty and obese frailty include grip strength, endurance, and physical activity. Our results align with previous studies indicating that obesity leads to significant reductions in grip strength [83,84] and physical activity [85–87]. Therefore, we suggest that the differences in grip strength and physical activity between obese frailty and pure frailty might be attributed to obesity. Contrary to prior studies [85–89], we did not find a differential effect of pure frailty or obesity on endurance. However, significant differences in endurance were observed between pure frailty and obese frailty. We hypothesize that this may result from a systemic metabolic disorder associated with frailty syndrome, which exacerbates the negative effects of obesity, thereby leading to decreased endurance in obese frailty.

### Overlapping and distinct biological phenotypes in pure frailty and obese frailty

This study is the first to report the overlapping and distinct biological phenotypes of pure frailty and obese frailty in skeletal muscle protein synthesis and degradation. Our data showed no significant difference in skeletal muscle protein synthesis between pure frailty and obese frailty, indicating an overlapping biological phenotype. Specifically, there were no significant differences in the expression of AKT, p-AKT, mTOR, and p-mTOR between the two groups. However, pure frailty, obesity, and obese frailty all resulted in decreased p-AKT expression with unchanged total AKT expression compared with the control. This suggests that these conditions reduce the rate of skeletal muscle protein synthesis by altering AKT activity [90]. Interestingly, the suppression of p-AKT expression by frailty syndrome and obesity was not additive in obese frailty. The specific mechanisms by which frailty syndrome and obesity together regulate p-AKT expression in obese frailty remain unclear. This may be due to the heterogeneity of skeletal muscle protein synthesis influenced by obesity [91] and/or the effects of frailty syndrome on multisystem metabolic disorders.

Our experimental results contradict prior studies. Pure frailty is often accompanied by whole-body metabolic changes that could influence the expression of Smad2/3, FoxO3a, and p62. For example, alterations in insulin-like growth factor 1 signaling, which can affect Smad2/3 protein accumulation, might contribute to the observed expression patterns in individuals with pure frailty [92,93]. It is also important to consider that the methods used to assess protein expression might influence the findings. For instance, if protein levels are measured instead of mRNA, post-transcriptional regulation could result in maintenance of protein levels despite changes in mRNA expression.

The expression of FoxO3a was significantly higher in both the obese frailty and obesity groups compared with the pure frailty group, likely due to obesity. While there was no significant difference in Smad2/3 and p62 expression between the obesity and pure frailty groups, it was significantly lower than in the obese frailty group, indicating that the difference is mainly caused by the combined effects of obesity and frailty syndromes. Obesity increases inflammation [94,95] and oxidative stress [96,97], which are risk factors for frailty syndrome, leading to variations in the expression levels of FoxO3a, Smad2/3, and p62 [31,98]. LAMP-2 is a crucial component of the lysosomal membrane that plays a vital role in autophagy [99], and its deficiency can lead to the accumulation of autophagic vacuoles in tissues, including skeletal muscle [100]. While aging is associated with a decline in autophagic flux due to decreased LAMP-2 expression, our data showed no significant changes in LAMP-2 expression in pure frailty, obesity, or obese frailty compared with the control group. However, obese frailty resulted in a significant decrease in LAMP-2 expression [101], suggesting that it may regulate LAMP-2 through mechanisms affecting Smad2/3, FoxO3a, and P62 due to the combined effects of frailty syndromes and obesity. Cathepsin L expression has been linked to inflammation [102] and autophagy [103], with overexpression causing tissue inflammation and underexpression leading to autophagosome accumulation [104]. Our findings indicated that pure frailty increases cathepsin L expression, while obesity and obese frailty do not, suggesting pure frailty might elevate cathepsin L due to muscle atrophy or systemic inflammation [102]. However, since the impact of obesity on cathepsin L remains unclear, we are currently unable to explain the differences in expression between pure frailty and obese frailty.

Similar levels of p-Smad2/3, p-FoxO3a, Beclin-1, ATG7, LC3-I, and LC3-II expression were observed in both pure frailty and obese frailty, indicating overlapping biological phenotypes in the regulation of skeletal muscle degradation. Frailty syndromes are often linked to aging, systemic metabolic disorders, inflammation, and oxidative stress [2,105,106]. These factors significantly affect the expression of p-Smad2/3 [107,108], p-FoxO3a [109,110], Beclin-1 [111,112], ATG7 [113,114], LC3-I and LC3-II [113–115]. We expected pure frailty to significantly alter these autophagy-related signaling molecules, but our results showed no such changes. Systemic metabolic disturbances caused by pure frailty might affect the expression of these molecular signals, requiring further investigation. The expression of autophagy molecules in obesity remains controversial, with inconsistent findings across studies. The results of some prior studies aligned with ours [101,116–118]. Since pure frailty and obesity did not significantly affect autophagy-related molecular signaling, obese frailty, as a combination of both conditions, also did not show significant changes in protein expression regulation.

## Limitations

Future studies using skeletal muscle maximal power, maximal muscle strength, and maximal contraction velocity might better reflect physical phenotypes that overlap or are distinct between pure frailty and obese frailty. Single-cell RNA sequencing technology could help elucidate gene expression differences between groups and characterize biological phenotypes that overlap or are distinct between pure frailty and obese frailty.

## Representative image of WB


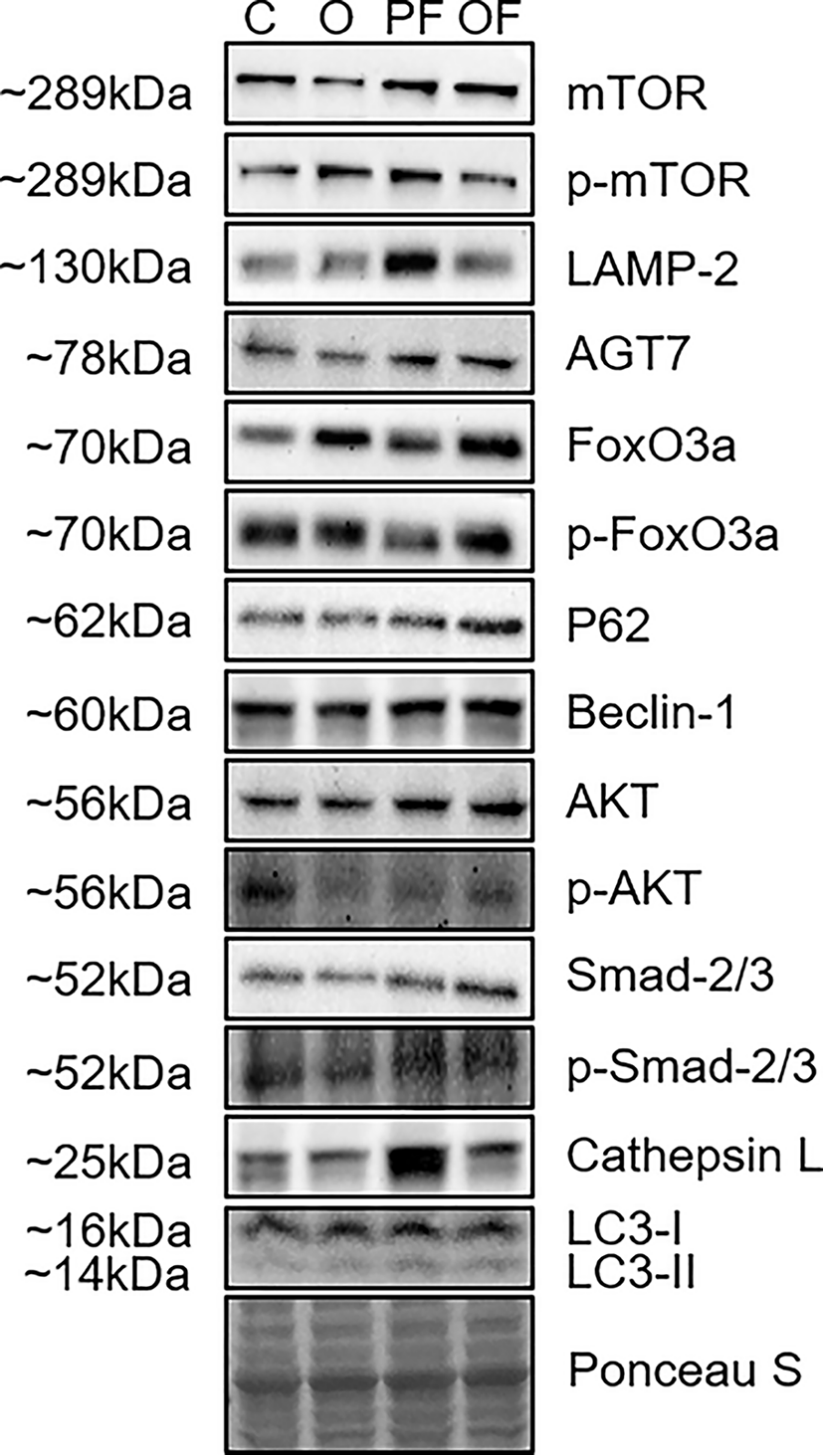


## Supplementary Material

supplementary files (S1)

## Data Availability

The data used to support the findings of this study are presented here. Any further data requirements are available from the corresponding author upon request.

## Abbreviations

SEM: Standard error of mean
TBST: Tris Buffered Saline with Tween 20
