# Supplementary material for: Overlapping and Distinct Physical and Biological Phenotypes in Pure Frailty and Obese Frailty
Source: Biosci Rep. 2024 Nov 6;44(11):BSR20240784. doi: 10.1042/BSR20240784 (PMC11554920; doi:10.1042/BSR20240784)
Supplement: supplementary files (S1) [file BSR-2024-0784_supp.pdf]

```
# Sample data: sets of biological markers for each group
pure_frailty = {"Grip", "walking speed", "activity", "p-AKT", "cath"}
obesity =    {"body weight", "lean mass", "fat", "fat in tissue", "Grip", "walking speed", "activity", "p-AKT", "foxo3a"}
frail_obesity = {"body weight", "lean mass", "fat", "fat in tissue", "Grip", "walking speed", "endurance", "activity", "p-AKT", "Smad2/3", "foxo3a", "ATG7", "p62"}

# Create a Venn diagram
venn3([pure_frailty, obesity, frail_obesity],
        ('Pure Frailty', 'Obesity', 'Frail Obesity'))

# Add a title
plt.title("Venn Diagram of Phenotype and Biology Overlapping/Distinct")

# Show the plot
plt.show()
```
